# Advice from the health insurer as a channelling strategy: a natural experiment at a Dutch health insurance company

**DOI:** 10.1186/s12913-018-3624-6

**Published:** 2018-11-06

**Authors:** Romy E. Bes, Emile C. Curfs, Peter P. Groenewegen, Judith D. de Jong

**Affiliations:** 1 NIVEL (Netherlands institute for health services research), Otterstraat 118-124, 3513 CR Utrecht, The Netherlands; 2 Open University, Valkenburgerweg 177, 6419 AT Heerlen, The Netherlands; 3 Maastricht University, Duboisdomein 30, 6229 GT Maastricht, The Netherlands

**Keywords:** Health insurance, Channelling, Preferred providers, Physiotherapists, Managed care

## Abstract

**Background:**

In a health care system based on managed competition it is important that health insurers are able to channel their enrolees to preferred care providers. However, enrolees are often very negative about financial incentives and any limitations in their choice of care provider. Therefore, a Dutch health insurance company conducted an experiment to study the effectiveness of a new method of channelling their enrolees. This method entails giving enrolees advise on which physiotherapists to choose when they call customer service. Offering this advice as an extra service is supposed to improve service quality ratings. Objective of this study is to evaluate this channelling method on effectiveness and the impact on service quality ratings.

**Methods:**

In this experiment, one of the health insurer’s customer service call teams (pilot team) began advising enrolees on their choice of physiotherapist. Three data sources were used. Firstly, all enrolees who called customer service received an online questionnaire in order to measure their evaluation of the quality of service. Enrolees who were offered advice received a slightly different questionnaire which, in addition, asked about whether they intended to follow the advice they were offered. Multilevel regression analysis was conducted to analyse the difference in service quality ratings between the pilot team and two comparable customer service teams before and after the implementation of the channelling method. Secondly, employees logged each call, registering, if they offered advice, whether the enrolee accepted it, and if so, which care provider was advised. Thirdly, data from the insurance claims were used to see if enrolees visited the recommended physiotherapist.

**Results:**

The results of the questionnaire show that enrolees responded favorably to being offered advice on the choice of physiotherapist. Furthermore, 45% of enrolees who received advice and then went on to visit a care provider, followed the advice. The service quality ratings were higher compared to control groups. However, it could not be determined whether this effect was entirely due to the intervention.

**Conclusions:**

Channelling enrolees towards preferred care providers by offering advice on their choice of care provider when they call customer service is successful. The effect on service quality seems positive, although a causal relationship could not be determined.

**Electronic supplementary material:**

The online version of this article (10.1186/s12913-018-3624-6) contains supplementary material, which is available to authorized users.

## Background

Several European countries such as Germany, Switzerland and the Netherlands, have reformed their health care system based on managed competition [1–4]. In health care systems that are based on managed competition, health insurers or other third parties, play an important role. They are supposed to prudently purchase care, on behalf of their enrolees. Health insurers also compete with each other, since enrolees are allowed to switch health insurers if they can get a better offer elsewhere. This is an incentive for health insurers to contract care providers based on price and quality of care in order to be able to offer attractive health plans. Additionally, they can compete by offering a high service quality [5]. Care providers compete with each other to be contracted by health insurers. However, the bargaining power health insurers have towards care providers depends largely on their ability to channel their enrolees towards contracted care providers [6, 7]. When they are successful in doing so, the market share of these contracted providers increases, which gives health insurers more bargaining power in negotiations with care providers.

There are different ways to channel enrolees towards contracted care providers. Those which have been researched include positive, and negative, financial incentives and quality incentives [8]. With positive financial incentives enrolees are given a discount, bonus or an exemption to paying a deductible when they use a preferred care provider [9]. Negative financial incentives mean that enrolees either have to pay a co-payment or they are not reimbursed, in full or in part, when they use a non-contracted care provider. Quality incentives may include, for instance, offering extended opening hours or a free health check at preferred care providers [10]. Positive financial or quality incentives, so called ‘soft’ incentives, are found to be successful. For instance, in the choice of a pharmacy, a quality certificate and extended opening hours were found to be effective channelling incentives [9, 10]. Yet, negative financial incentives are shown to be more effective. This is also the most implemented type of incentive [8]. However, multiple studies show that enrolees feel negative about such incentives and do not want their health insurer to limit their choice of care provider [11–13]. This has led to the so called managed care backlash in the US, a collective resentment against managed care [14]. For this reason, health insurers in the Netherlands are reluctant to implement selective contracting [15]. Ideally, health insurers channel their enrolees towards preferred care providers in a positive manner, oriented towards the service, and in a way that does not emphasise a limitation in care provider choice. Therefore, it is important for health insurers to deliver a high quality of service since this can improve customer loyalty and satisfaction with the company [16–18].

Health insurers are contacted by their enrolees many times every day. This provides an opportunity for health insurers to offer a good quality of service and improve the relationship with their enrolees [19, 20]. A Dutch health insurance company saw these calls as an opportunity to channel enrolees towards preferred care providers, while, at the same time, increasing customer satisfaction. The idea is to channel enrolees to a preferred care provider when, for instance, they ask a question about the reimbursement of care. After answering the question, the employee has a chance to ask the enrolee if he or she has already chosen a specific care provider and to offer advice on which care provider to choose. This is offered as an extra service during the phone call. The health insurance company tried this out with one of their customer service call teams. Because of the prolonged collaboration between the research institute NIVEL and this health insurance company, the researchers were informed about this initiative and were given the opportunity to collaborate with the health insurance company and to design this study to measure the effects of this natural experiment. The research questions we aimed to answer were: Is it possible to channel enrolees towards preferred care providers by giving them free advice when they call customer service and what is the effect of this service on enrolees’ rating of the service quality?

### Context

A health care system based on managed competition was implemented in the Netherlands in 2006. However, the implementation of selective contracting by health insurers has proceeded slowly since health insurers have been reluctant to implement selective contracting and negative financial incentives. This is because they feared their enrolees would distrust them and change insurers. Enrolees in the Netherlands are allowed to switch health insurers every year during a specific period [21]. In 2014, article 13 of the Health Insurance Act was to be revised in order to allow health insurers to determine the level of reimbursement for non-contracted care providers. However, this was rejected by the First Chamber of parliament, which resulted in health insurers still being obliged to reimburse at least 75% of the costs of non-contracted care providers [22]. This has made channelling enrolees towards contracted care providers more difficult [23]. Some health insurers, however, still offer restrictive health plans where non-contracted care providers are reimbursed up to 75%. However, enrolees do not often choose these types of health plans [24]. Additionally, it is the younger and healthier enrolees who are more likely to choose these restrictive health plans [25]. This means that care providers hardly lose any business because of these contracts since most enrolees who use care, have health plans with a free choice of provider. This has negative consequences for the bargaining power of health insurers in relation to care providers. Thus, although it is important for health insurers to be able to channel their enrolees towards preferred care providers, they are hardly able to do so. Research showed that qualitative incentives can also have an effect upon enrolees’ care provider choice, although to a lesser degree than negative financial incentives. There was hardly any information found about whether health insurers in the Netherlands currently use these types of incentives.

In addition to offering a good quality of care for a good price, it is also very important for health insurers to create a good relationship with their enrolees in order to build loyalty. A way to channel enrolees towards preferred providers, while maintaining or building a good relationship with enrolees is therefore essential. The current study investigates whether it is possible to channel enrolees towards preferred care providers when they call customer service and to measure how far this affects enrolees’ assessment of the quality of service.

## Methods

### Setting

The setting for this study was a large Dutch health insurance cooperative (VGZ), which is one of the four market leaders in health insurance in the Netherlands. Their customer service is called by many enrolees for a variety of questions. Every week 40.000–100.000 calls are handled. This is an opportunity for them to give, when applicable, enrolees advice on the quality of care providers and channel them towards good quality, preferred, care providers. They focused on physiotherapists, since the health insurance company had recently implemented a system to determine the quality of the performance of physiotherapists [26]. This quality measure is based on effectiveness, in which the physiotherapist who cures conditions with the fewest number of treatments, is considered the best. This method provides insight in the extent to which a practice deviates from the number of expected treatments per patient, corrected for characteristics of the patients. This was conducted on a national level and thus includes all physiotherapists. There are four tiers, the first tier being the most effective, the fourth the least effective. The goal of the insurance company is to channel enrolees towards physiotherapists in tier one or two. For the enrolees, there is no financial incentive to use a more effective physiotherapist. They do, however, benefit from undergoing fewer treatments, since enrolees are entitled to a limited amount of treatments per year. Not all enrolees have the same benefits for physiotherapy, since this care is included in voluntary additional insurance packages. All benefits are restricted to a certain amount, which depends on the insurance package enrolees chose. Since physiotherapy is only reimbursed to a certain amount, enrolees know they have to check their budget before they use care.

One of the customer service call teams, comprising 14 employees, was chosen to try out the new channelling method which we were studying. They received 5 h of training so they could recognise when and how they could offer advice about the care provider choice of enrolees and how to explain this to enrolees. For instance, for all calls about physiotherapy, employees were supposed to ask their customers whether they had already chosen a physiotherapist and whether they would like advice about the quality of physiotherapists in the area where they live. The employees were given access to information about the effectiveness of physiotherapists on a local level. So when they received a call from an enrolee about physiotherapy, they were able to identify all the physiotherapists in the neighbourhood where the enrolee lived together with information about their effectiveness, which they could in turn explain to the enrolee. Of course, besides effectiveness, there are other aspects of quality which enrolees may find important, such as how patients are treated and how physiotherapists communicate with their patients. However, the employees only had information on effectiveness of the physiotherapists - that is which physiotherapist can help to cure conditions using the fewest number of treatments. It was found that enrolees find this aspect of quality important [27]. The pilot team started offering this extra service in August 2016. Normally, customer service call teams start their week with a short meeting. The pilot team had these meetings three to four times during the week to discuss progress and any difficulties. After the initial period, in October 2016, a session was held with the employees in order to evaluate the calls and to make sure calls were logged correctly by the customer service employees. After this meeting the official data collection started.

### Design

The study can be seen as a natural experiment. A natural experiment is an empirical study in which individuals (or clusters of individuals) exposed to the experimental and control conditions are determined by nature or by other factors outside the control of the investigators. The health insurer conducted the experiment and enrolees are randomly assigned to customer service employees.

**Fig. 1:**
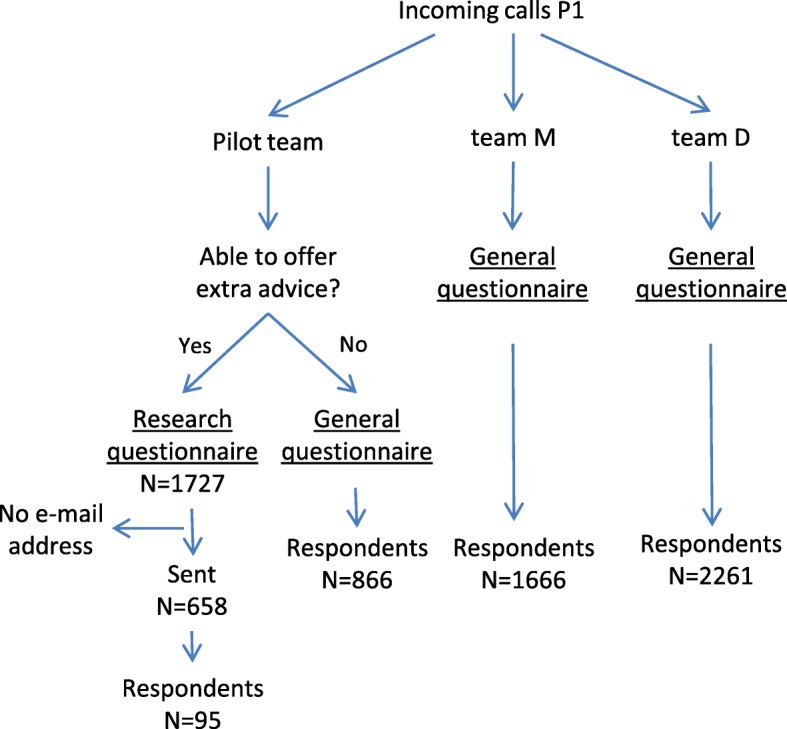
Questionnaire scheme and overview of the response during P1

We used three data sources for this study. Firstly, data were used from questionnaires sent to enrolees who had contact with customer service. Two different questionnaires were sent. At this health insurance company, all enrolees who call customer service, receive a short questionnaire afterwards via e-mail about the service they received (general questionnaire, Fig. 1). This questionnaire has been developed by the health insurer. For enrolees who, during the study period, had contact with an employee of the pilot team who offered extra advice on the choice of care provider, a specific questionnaire was developed by the researchers (research questionnaire, Fig. 1). In the research questionnaire, service quality was measured in the same way as in the general customer service questionnaires. This enabled us to compare the results from different customer service teams and, thus, to analyse the service quality scores of the pilot team. Other questions were added in the research questionnaire in order to find out why customers were open to advice or not, how they felt about the advice and if they intended to follow the advice to go to the preferred care provider. All questionnaires were sent out automatically by e-mail within a few days of the phone call. Secondly, customer service employees logged every phone call, registering the enrolee who called, the subject of the phone call and the answer they gave to the enrolee. Employees of the pilot team also specifically registered if they were able to offer extra advice to the enrolee and whether the enrolee accepted this advice. Furthermore, they marked the unique identification code of the physiotherapist they referred the enrolee to.

Thirdly, the logged data of the employees was combined with data from the insurance claims of enrolees that have received an advice in order to see if enrolees chose the recommended care provider or not. This was performed by employees of the health insurer.

Quality assessment of the collected data was only possible to a very limited degree. The questionnaires were checked for completeness and questionnaires with missing values on the outcome variables were left out. We had no way to check the fidelity of the experiment. Employees logged whether or not they had given advise, but whether they had actually done what they wrote in the log could not be checked. Finally, we assume that the link to claims data was correct, as this is part of the core administrative processes of the health insurance company.

### Outcome measures and analyses

#### Channelling

In order to see whether this method can be a successful channelling method two outcome measures were used. Firstly, we asked in the questionnaire whether enrolees were willing to follow the advice of their health insurer with the question: ‘How likely is it that you will follow the advice of the employee?’ This was measured on a scale of one (not likely) to five (very likely). Secondly, we could see whether enrolees actually follow the advice of the health insurer when the registration data of employees is combined with the claims data. The data that is logged by the employee for each call contained information on whether a specific advice was given and which care provider was recommended. This information was linked to the claims data to see whether enrolees had actually visited the recommended physiotherapists.

#### Service quality

To measure the effect of the intervention on the quality of service, we measured customers’ evaluation of the phone calls. The quality of service was measured in the questionnaires by asking: ‘Could you grade the conversation you had with the employee?’ Respondents could indicate a number from one to ten, where one is the worst and ten is the best score. Half way through the research period, in the middle of January 2017, the insurance company revised their general customer service questionnaire by altering this question to: ‘Could you grade the employee you had contact with?’ This was still on a scale from one to ten. Although the questions are very similar, they are not the same. Therefore, we also added this new question to the research questionnaire. In this way we were able to check if we could use this variable as a proxy for the original question on the quality of service. In the cases where both versions of the service quality question were answered (*N* = 54) the questions were compared. The correlation between the questions was .89 (*p* < 0.001). Furthermore, we compared the means of both questions and found little to no differences. Therefore, we chose to use the new question as a proxy for the quality of service in the cases where the original question was removed. Furthermore, in the research questionnaire, enrolees who accepted the offered advice were specifically asked to rate the advice they were given.

**Fig. 2:**
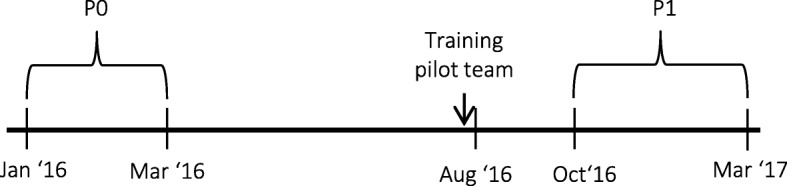
Overview of the timeline

In order to measure how the pilot team scored on service quality compared to regular customer service, we compared the scores of the pilot team on the quality of service with two other teams over two time periods: period P0 (January – March 2016) and period P1 (research period, from 20 October 2016 – March 2017) (Fig. 2). For the pilot team, the research questionnaire data was merged with the data from regular customer service questionnaires in order to get an overall score for the quality of customer service. The other two teams were chosen to be as similar as possible to the pilot team in terms of the subjects of the calls they receive and the clients that call them – that is they were customers from the same health plans.

#### Statistical analysis

The data was hierarchical, since calls were clustered within employees and employees were clustered within teams. Multilevel linear regression analyses were conducted using Stata 14. The dependent variable was the service quality rating and the independent variables were the team (pilot team/customer service team M/ customer service team D) and the period (P0/P1). The model included two levels. The enrolees were the lowest level and the employees the higher level, to correct for the cluster effect of this hierarchical data. To assess whether the pilot team performed better compared to the other teams on P1 an interaction term of team and period was added.

On P0, the teams did not exist in the current formation. The employees in the pilot team and the two similar teams did work as customer service employees, however in different team formations. Around April 2016, the current teams were formed. The composition of the teams stands apart from the intervention. In order to have a score for the current teams during P0, the service quality scores during P0 of all individual employees who are currently in the pilot team and the two similar teams were used.

## Results

**Fig. 3:**
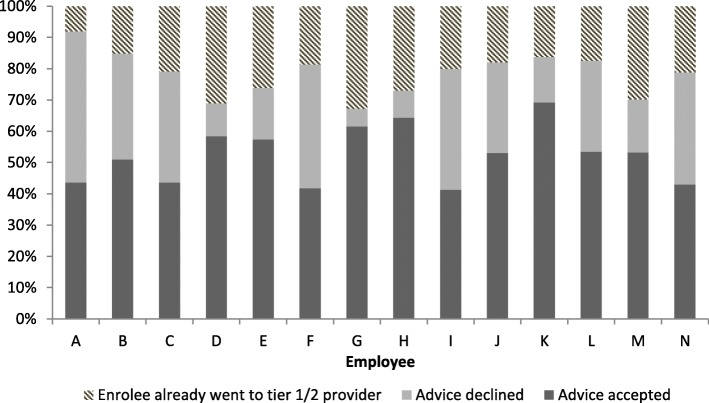
Differences in offering advice between employees of the pilot team

During the research period (24th October 2016 – 31st March 2017) advice was offered by the pilot team in 1727 calls - that is 5% of the total number of calls they handled in this period. The team logged 51.3% of the 1727 calls as conversations where the enrolee was open to the advice that was offered. In 27.5% of the calls advice was offered, but the enrolee declined it. In 21.2% of the cases enrolees already went to a physiotherapist from tier one or two. Furthermore, most of the 1727 calls (93.6%) were categorised by employees under two subject categories; (1) the enrolee had a question about the reimbursement of care they had not yet received; and (2) an enrolee called to inform them about a change in their personal information, such as a change of address. We found considerable differences between employees. Some employees were able to offer advice more often than others and some employees logged relatively higher numbers of advice as accepted by the enrolees compared to others. The differences between employees still existed even after the session on 20th October where these kinds of differences were discussed (Fig. 3).

Unfortunately, it turned out that the insurance company did not have e-mail addresses of all their enrolees. Therefore, only 658 of the 1727 enrolees received a research questionnaire. Ninety-five research questionnaires were completed, a response rate of 14,9% (Fig. 1). This is a low response rate, however, it was somewhat higher than the response to the standard customer service surveys (12,3%) this insurance company sends to their enrolees.

The average age of the 95 respondents to the research questionnaire is 56,7 years (SD 12,7), and 71% are female. All respondents were asked if they accepted the advice the employee offered them. Twenty-eight respondents indicated they had not been offered advice or did not remember. Thirty-two respondents indicated they had accepted the advice and 35 indicated they had not. Most indicated that the reason not to accept the advice was that enrolees had already chosen a care provider (*N* = 19). Other answers were, “I do not want advice from my health insurer” (*N* = 4), and, “it is not yet necessary to use a care provider” (*N* = 4). Compared to the data which was logged by the employees, data from the questionnaire shows a lower percentage of calls where enrolees accepted the advice offered. Also, many enrolees indicated they had not been offered advice about their choice of care provider. Employees apparently sometimes registered that an enrolee had accepted advice while the enrolee did not see it that way.

### Channelling

Respondents who were offered advice and indicated they had accepted it (*N* = 32) were asked if they plan to follow up on the advice. Only 16 respondents answered this question, of which 13 indicated a 4 or a 5, meaning that they were likely, or very likely, to follow it. Since this is a very small group, we also looked at the data that was collected during the initial period (August 2016–20 October 2016). This resulted in 35 respondents answering this question. Of these respondents 31 indicated a 4 or a 5, meaning that they were likely, or very likely, to follow it. This confirms the results from the research period. However, the number of responses is still very low.

The logged data from the employees shows that 484 enrolees were given a specific advice on which physiotherapist to choose. Claims data showed that a large part of these enrolees did not, or had not yet visited, a physiotherapist (*N* = 193, 39,9%). Of all enrolees who were given specific advice on which physiotherapist to choose and who also went to a physiotherapist after the call (*N* = 237), 45% followed the advice that was given (*N* = 106). Of these 106 enrolees that followed the advice, 60% did not have a physiotherapist before, 26% switched, and 14% already went to the advised physiotherapist before the call.

### The quality of service

Respondents to the research questionnaire (*N* = 95) rated the general quality of service on average with a 7.8 (SD 1.4). Respondents who indicated they accepted advice from the employee (*N* = 32) rated the advice on average with an 8.3 (SD 0.9). Forty-two of the 65 respondents who indicated they were offered advice partly or completely agreed with the statement, ‘I appreciated it that the employee offered me advice on the choice of care provider’.

In Table 1 the scores for the quality of service for each team are shown during P0 (January to March 2016) and during the research period P1 (20th October 2016 to March 2017) (for more details see Additional file 1: Appendix 1). During P0, the pilot team scored the lowest on service quality compared to the other two teams. During P1, all teams had improved their scores. However, the pilot team had improved more compared to the other two teams.

**Table 1:** Service quality scores for the teams during P0 and P1

	P0 Jan-Mar ‘16	N	Age (SD)	P1 20 Oct ‘16– Mar ‘17	N^a^	Age (SD)	Difference in service quality scores
Pilot team	7.44	1037	57,9 (13,4)	8.14	959	57,2 (12,6)	+.70
Team M	7.47	998	57,7 (12,9)	7.67	1659	59,4 (12,0)	+.20
Team D	7.56	848	58,7 (13,0)	7.71	2258	60,2 (12,9)	+.15

^a^ N during P1 differs slightly from numbers in Fig. 3, because of a small number of missing values on the question of the quality of service

Multilevel regression analyses showed that the increase of the pilot team from P0 to P1 is significant (*p* < .001). Furthermore, it showed that the pilot team significantly increased their score more compared to both other teams (both *p* < .001).

We know from the registration data that most incidents of advice were given when enrolees asked questions about the reimbursement of care they had not yet consumed and when they reported a change in their personal information, such as a change of address. Therefore, we decided to repeat the analyses while selecting only these subject categories for all three teams. In Table 2, only the scores for the quality of service for these subject categories were presented for P0 and P1. This shows that for these subjects the quality of service overall was rated slightly higher compared to the scores in Table 1. Still, the pilot team improved the most compared to both the other teams.

**Table 2:** Service quality scores for the teams during P0 and P1 for caller categories ‘expectations’ and ‘changes’^a^

	P0 Jan-Mar ‘16	N	P1 20 Oct ‘16– Mar ‘17	N	Difference
Pilot team	7.84	274	8.32	576	+.48
Team M	7.73	266	7.90	584	+.17
Team D	7.98	235	7.90	859	−.08

^a^ The sample was created by selecting only the calls that were classified in the categories ‘expectations’ or ‘changes’ as these were the categories that most of the incidents of advice were given

Multilevel regression analyses showed that the increase in the quality of service of the pilot team from P0 to P1 is significant (*p* < .001). Furthermore, the pilot team score improved significantly more compared to customer service team D (*p* = .003). The difference in improvement between the pilot team and customer service team M is not significant. Customer service team M did not significantly improve more compared to customer service team D.

## Discussion

This study aimed to investigate the possibility of channelling enrolees towards preferred care providers by offering them advice on care provider choice when they call customer service. The idea is that this way of channelling is more positive compared to negative financial incentives. In addition, as it is offered as an extra service, it may even increase enrolees’ evaluation of the quality of customer service. The results show that offering advice on the choice of a care provider when enrolees call about something else is most applicable at certain moments. These are when enrolees have questions about the reimbursement of care they may want to consume in the future or when they want to report a change in their personal records such as a change of address. In general, enrolees are positive about their insurer offering advice. Giving advice is also an effective channelling method, since it was found that 45% of enrolees who went to see a physiotherapist after they were given advice, actually go to the recommended physiotherapist. In addition, results show that service quality ratings increased.

### Channelling enrolees to preferred care providers

This way of channelling enrolees to preferred care providers has not been studied before. The results seem very promising since a large part of the enrolees that go to see a physiotherapist after they received advice, followed the advice. However, whether or not it is possible to channel enrolees to preferred care providers during a call to customer service may depend on different factors. Some enrolees may be opposed in principal to receiving advice from their health insurer about the quality of care providers. Previous research from the Netherlands showed that almost 55% of enrolees do not welcome advice about their choice of care provider from their health insurer. Respondents mostly indicated that they wanted, and/or were able, to choose a care provider themselves, they think their health insurer has its own interests at heart or is not objective, and they would rather accept advice from their GP [28]. These reasons point towards a trust issue. Health insurers are generally not very highly trusted by enrolees [28, 29]. However, trust in a company (institutional trust) is usually lower compared to trust in a person (interpersonal trust) [30]. Therefore, it may be different when an employee personally gives advice instead of the health insurer in general, for instance on their website or by sending a letter. However, the current study showed that only a small part of the enrolees who did not want advice indicated they did not want advice from their health insurer. Most of them did not, or did not yet, need a care provider and therefore declined the offered advice. Furthermore, the advice was given for free and whether one followed the advice or not did not have any negative financial consequences for enrolees.

When employees gave advice they also had the chance to explain the reason why a certain physiotherapist was better than others. Research showed that information about the selection of care providers is important in order to accept the negative consequences of selective contracting (Bes RE, van Erp KJPM, Curfs EC, Groenewegen PP, de Jong JD: Acceptance of selective contracting in health insurance: importance of information, submitted). Therefore, it is very important that the health insurer is able to explain to enrolees why a certain care provider is preferred, or delivers better quality of care, compared to other care providers. If such processes are explained well, it is possible that enrolees are more willing to accept the advice they are offered (Bes RE, van Erp KJPM, Curfs EC, Groenewegen PP, de Jong JD: Acceptance of selective contracting in health insurance: importance of information, submitted). It is important that enrolees agree with the health insurer on aspects of the quality of care they use. If enrolees do not care about the duration of the treatment, but more about the communication skills of the physiotherapist or the convenience of the location, it may not work. It is likely that most enrolees are interested in having an effective physiotherapist, since many enrolees followed the advice in the current study.

Previous research has shown that it is very hard to channel enrolees towards preferred care providers when they already have a relationship with another care provider, even when a better alternative is available [31]. This is called status quo bias. If enrolees are currently being treated by a physiotherapist, it is unlikely they will follow the advice of the health insurer if the employee advises another physiotherapist. An exception to this can be when enrolees are not satisfied with their current care provider. This may even make it easier to channel them towards a preferred care provider. This is also found in the current study. Most enrolees who followed the advice had not chosen a physiotherapist yet and were therefore relatively easy to channel towards a preferred provider. However, there were also some enrolees who switched physiotherapists after the advice. It would be interesting to look further into why they switched, how many treatments they already had and whether they were unhappy with their current provider. Given that it seems to be important for successful channelling that enrolees have not already chosen a care provider, it will be hard to channel enrolees to preferred GPs, dentists or other care providers with whom they already have a relationship. Boonen et al. found that in 2009, in the Netherlands, the willingness of enrolees to listen to advice from the health insurer about their choice of care provider is greatest for hospitals and pharmacies. For GPs and dentists this willingness is the lowest and for physiotherapists this is a little bit higher than GPs but much lower than pharmacies and hospitals. This suggests that advising enrolees on hospitals and pharmacies may be very interesting to investigate next. It is expected, however, that enrolees will be hard to channel if they already have a relationship with a specialist at a specific hospital, for instance when enrolees have a chronic condition.

### Service quality

It is important to note that the results show that ratings of the quality of service of the pilot team are not significantly different compared to the other customer service teams. Enrolees generally dislike their health insurer interfering with their choice of care provider. A successful channelling method that does not harm the quality ratings is therefore already very valuable. However, the idea was that offering enrolees help with choosing a care provider as an extra service, whether enrolees accept it or not, could improve enrolees’ rating on service quality. Research by Rafaeli et al. shows that offering personalised information and giving explanations lead to a higher evaluation of customer service interactions [32]. The current study points to a possible improvement in the quality of service. However, since the teams in their current form did not exist in T0, the improvement in service quality scores could also be caused, at least partly, by the change in the composition of the teams. Although the job of customer service call centre employee is very individual, there are team meetings and, thus, interactions between team members. Furthermore, the pilot team was very happy with their new task. They really liked giving enrolees extra advice and they liked being informed by other departments that purchase care and assess the quality of care providers. An employee satisfaction survey showed high scores for the pilot team. Furthermore, the pilot team had more meetings during the week compared to other teams. A positive feeling and satisfaction with their job is likely to have had a positive influence upon other calls the pilot team handled. Such a situation can be referred to as the Hawthorne effect. To be more certain of the effect on service quality scores, the pilot team should be followed longer to see what happens when the new task is not new anymore, but more integrated into their standard work process. It is also possible to use a control group in which the same amount of attention is given but who are not given the extra task of giving advice on the choice of care provider.

High service quality ratings are very important for health insurers since these could lead to more trust in the company [33]. Trust was found to lead to customer loyalty [34] and was found to play an important role in the acceptance of selective contracting [35]. Also, when the health insurer enjoys greater trust, all information the health insurer provides may also be trusted more. This could be very important with regard to the intention of enrolees to follow advice from the health insurer [8].

### Strengths and limitations

A strength of this study is the unique opportunity to evaluate a channelling experiment designed by a large Dutch health insurance company. Furthermore, we were able to use different data sources. It was possible to find out exactly how far enrolees followed up on the advice that was given. However, a few limitations need to be discussed. The response rate to the questionnaires was low. This goes for the research questionnaire, but of course also for the other questionnaire data we used to compare the service quality scores of the pilot team to other teams. Unfortunately, the health insurer has no insight into response bias, since the questionnaires are filled in anonymously. When we compare the data from the research questionnaire to the claims data, we find that in the research questionnaire, almost all respondents state they are willing to follow the advice of the employee. This is more than the 45% who actually followed the advice according to the claims data. Thus, it seems that enrolees who fill in the questionnaire may be more positive about their conversation with the employee, compared to enrolees who do not. Furthermore, we had no background characteristics available for the general customer service questionnaires, therefore we were not able to correct for respondent characteristics such as age and sex in the multilevel analyses. However, the two teams that were chosen as control groups were as similar as possible to the pilot team, with respect to the enrolees they help and the types of questions they get asked. Therefore, the assumption was made that the enrolees did not differ in their background characteristics.

### Policy implications

The results of this study indicate that it is possible to channel enrolees by offering advice to those who call customer service. Since enrolees experience the offered advice as positive, this may be a good alternative to using financial incentives to channel enrolees towards preferred providers. However, compared to implementing financial incentives, this method does not reach all enrolees, but only the enrolees who call customer service. It is therefore crucial that when this service is implemented, health insurers promote this service among all their enrolees. Furthermore, the timing in the care chain is very important. If enrolees have already chosen a provider they are less likely to be open to advice on their choice of care provider.

There were great differences between employees in how many times they offered advice and in the percentages of the advice that was accepted or declined. This shows that the extra task of offering advice is quite difficult to generalise. It depends on the skills of the employee in recognising an opportunity to offer extra advice. Thus, this channelling method may require different competences from call centre employees. Higher educated employees may be necessary. This needs to be taken into account when implementing such a channelling strategy on a larger scale. Also, the calls may take longer. This was hard to measure during the current study, but the total handling time, based on the average of all calls of the pilot team, was about the same compared to the other two teams. However, advice was offered in only 5% of the total number of calls. If this share increases, once this channelling method is expanded to other care provider types, then the impact upon call handling time may be greater. However, if the channelling strategy is successful, then this will increase the health insurers’ bargaining position with respect to care providers, which is supposed to lead to lower costs for the health insurer. This may compensate for extra costs in personnel and handling time.

Although it is not clear how far the improvement in scores for service quality can be attributed to the calls where extra advice was given, an improvement in service quality was found. From the perspective of the health insurer it may not even be that important what exactly caused the improvement in service quality. However, if it was caused by the Hawthorne effect, thus by simply being a part of the study and thus the job receiving more attention, then it is possible that the effect will wear off after some time. It is important to note that at least the ratings for quality did not go down. The study showed that channelling enrolees by offering advice is successful and that it does not provoke a negative response from enrolees. This is a very important finding, since this is the case with negative financial incentives.

### Scientific implications

This study contributes to the literature on the effectiveness of channelling methods. Thus far this method of offering advice on care provider choice to enrolees when they call customer service has not yet been researched. One study from the US reported a natural experiment where enrolees were offered a telephone service to help them choose a care provider [36]. However, this service was provided by the employer, not the insurer, and relies on enrolees seeking help with choosing a care provider, whereas this method specifically offers advice to enrolees who did not seek help with this choice. Furthermore, this study shows that even when trust in health insurers is not very high, many enrolees may still be open to free advice from their health insurer. This may be because trust in the individual employee of the health insurer is higher than trust in the health insurer in general. Furthermore, this channelling method may even improve trust in the health insurer since it was found that improvement in the quality of service may lead to greater trust in the company [33].

## Conclusion

This study shows that channelling enrolees towards preferred care providers by offering them advice on care provider choice when they call customer service can be successful. Since enrolees are very negative about financial incentives, this may be a good alternative channelling method. It is hard to draw solid conclusions from data on service quality, but the results seem positive.

## Additional file

Additional file 1: Appendix 1. Multilevel analyses. (XLSX 15 kb)

## Abbreviations

GP: General practitioner
NIVEL: Netherlands institute of health services research
